# A study protocol for a predictive algorithm to assess population-based premature mortality risk: Premature Mortality Population Risk Tool (PreMPoRT)

**DOI:** 10.1186/s41512-020-00086-z

**Published:** 2020-11-04

**Authors:** Laura C. Rosella, Meghan O’Neill, Stacey Fisher, Mackenzie Hurst, Lori Diemert, Kathy Kornas, Andy Hong, Douglas G. Manuel

**Affiliations:** 1 Dalla Lana School of Public Health, University of Toronto, 155 College St, 6th floor, Toronto, Ontario M5T 3M7 Canada; 2 Public Health Ontario, 480 University Avenue, Suite 300, Toronto, Ontario M5G 1V2 Canada; 3 Institute for Clinical Evaluative Sciences, 2075 Bayview Ave, Toronto, Ontario M4N 3M5 Canada; 4 University of Oxford, The George Institute for Global Health, Nuffield Department of Women’s & Reproductive Health, Hayes House, 75 George Street, Oxford, OX1 2BQ UK; 5 Ottawa Hospital Research Institute, Ottawa, Canada; 6 Statistics Canada, Ottawa, Canada; 7 Department of Family Medicine, University of Ottawa, Ottawa, Canada; 8 School of Epidemiology and Public Health, University of Ottawa, Ottawa, Canada; 9 Bruyère Research Institute, Ottawa, Canada

**Keywords:** Premature mortality, Prediction model, Study protocol, Weibull model, Population health

## Abstract

**Background:**

Premature mortality is an important population health indicator used to assess health system functioning and to identify areas in need of health system intervention. Predicting the future incidence of premature mortality in the population can facilitate initiatives that promote equitable health policies and effective delivery of public health services. This study protocol proposes the development and validation of the Premature Mortality Risk Prediction Tool (PreMPoRT) that will predict the incidence of premature mortality using large population-based community health surveys and multivariable modeling approaches.

**Methods:**

PreMPoRT will be developed and validated using various training, validation, and test data sets generated from the six cycles of the Canadian Community Health Survey (CCHS) linked to the Canadian Vital Statistics Database from 2000 to 2017. Population-level risk factor information on demographic characteristics, health behaviors, area level measures, and other health-related factors will be used to develop PreMPoRT and to predict the incidence of premature mortality, defined as death prior to age 75, over a 5-year period. Sex-specific Weibull accelerated failure time models will be developed using a Canadian provincial derivation cohort consisting of approximately 500,000 individuals, with approximately equal proportion of males and females, and about 12,000 events of premature mortality. External validation will be performed using separate linked files (CCHS cycles 2007–2008, 2009–2010, and 2011–2012) from the development cohort (CCHS cycles 2000–2001, 2003–2004, and 2005–2006) to check the robustness of the prediction model. Measures of overall predictive performance (e.g., Nagelkerke’s *R*^2^), calibration (e.g., calibration plots), and discrimination (e.g., Harrell’s concordance statistic) will be assessed, including calibration within defined subgroups of importance to knowledge users and policymakers.

**Discussion:**

Using routinely collected risk factor information, we anticipate that PreMPoRT will produce population-based estimates of premature mortality and will be used to inform population strategies for prevention.

## Background

Premature mortality is an indicator that represents the concept of an unfulfilled life expectancy and is meaningful in the context of public health as premature deaths are largely amenable to targeted policy and programmatic interventions [1, 2]. As such, premature mortality is an important indicator of population health that has been used to assess health system functioning and to identify areas in need of targeted health system intervention. The Canadian Institute of Health Information defines Canadian premature mortality using an age cut-off of 75 [3], which is consistent with the age range adopted to capture premature mortality in other industrialized countries [4–6]. Premature mortality is an important metric for evaluating which population sub-groups are benefitting from public health, medical care, and health policy and which groups are being left behind. For example, in recent years, premature mortality rates have stagnated in Canada [7] and appear to be increasing in the USA [8] and Europe [9, 10] after historically experiencing steady declines. Gaps in premature mortality across socioeconomic status are widening both in Canada [11–15] and internationally [16–18]. Additionally, premature mortality rates can be used to compare population health status between groups, regions, and health systems [4]. As one of the foremost goals of public health, reductions in premature mortality have been identified by the United Nations sustainable development goals for 2030 as a major priority that focuses on prevention and promotion of health and well-being [19].

Health system decision-makers are increasingly interested in using population-level data to strategically inform which interventions may result in the greatest benefit to the population [20, 21]. The ability to predict population subgroups or geographic regions with high risk of future premature mortality is a considerable advantage from a public health planning perspective and can facilitate initiatives that promote equitable health policies and effective delivery of public health services. The majority of existing research using population health survey data has focused on characterizing risk factors for all-cause mortality, including in Canada [22] and the UK [23]. Several characteristics that are commonly associated with elevated premature mortality risk include disease indicators (e.g., chronic disease), health behaviors (e.g., smoking, physical inactivity, alcohol consumption, and poor diet), socioeconomic measures (e.g., income), and psychosocial factors (e.g., self-reported health status) [22–26]. Previous research suggests that well-known and modifiable risk factors explain a large amount of premature mortality emphasizing the importance of population-based efforts to reduce the burden of premature mortality [27].

To date, the majority of prediction models have focused on all-cause mortality [28], all-cause mortality in defined population subgroups (i.e., infant mortality, maternal mortality, trauma patients) [29–31], or use data sources (i.e., electronic health records, biological specimens) that are not publicly available [32]. To our knowledge, no population-level risk prediction algorithm, using routinely collected public available data, has been developed for premature mortality. To guide population-level preventative action, we propose the development and validation of a population-level risk prediction algorithm, the Premature Mortality Population Risk Tool (PreMPoRT). This tool will be developed using a multivariable modeling approach, linking self-reported risk factor data collected by a large population-based community health survey in Canada linked to vital statistics databases. This study protocol is presented to prespecify the predictive variables and analytic plan to increase the robustness, validity, and transparency of the model.

## Methods

### Data sources

PreMPoRT will use national population-based survey data from the Canadian Community Health Survey (CCHS) linked to the Canadian Vital Statistics Database (CVSD). The CCHS is a cross-sectional survey conducted by Statistics Canada that began in 2000 that collects information on health status, health care utilization, and health determinants among the Canadian population 12 years and older [33]. The CCHS features a multistage, stratified cluster survey design where the household is the final sampling unit. Overall, the CCHS represents just over 98% of the Canadian population with an average response rate of 80.5%. Certain population subgroups are excluded from the sampling frame including people living on First Nation Reserves and Crown Lands, institutional residents, and full-time members of the Canadian Forces. The survey was conducted through interviews by telephone and in person, and all survey responses were self-reported. All self-reported predictors for PreMPoRT will be obtained from the CCHS. Details of survey methodology for the CCHS have been previously published elsewhere [33].

### Study design

PreMPoRT will include two sex-specific models that will be derived and validated using population-based provincial data in Canada available through Statistics Canada [34]. All analyses will be sex stratified given important sex differences related to mortality and risk factors [13, 35]. All CCHS respondents in Canadian provinces from the first six cycles, who consented to have their responses linked to the CVSD, will be included. The derivation cohort will consist of the first three cycles of the CCHS—cycles 1.1 (conducted 2000–2001), 2.1 (conducted 2003–2004), and 3.1 (conducted 2005–2006). External validation will be performed using the CCHS cycles from 2007–2008, 2009–2010, and 2011–2012. The external validation will examine the prediction models’ performance in the same source population but using different individuals surveyed over a different time period. For both development and validation cohorts, respondents will be excluded if they were under the age of 18 or older than 74 years as of the CCHS interview date. Respondents who are pregnant will also be excluded due to the inability to accurately ascertain baseline body mass index (BMI). Among the small proportion of survey respondents who had multiple CCHS survey responses (< 2%), the earliest record after the age of 18 years will be used. Bootstrap replicate survey weights will be incorporated for development and validation to account for the CCHS’s complex survey design and to produce estimates that reflect the population demographics of Canada. Sampling weights will be used during the regression estimation such that the beta coefficients generated account for the sampling design and non-response through a weighting procedure. Variance estimates will be calculated as recommended by Statistics Canada using bootstrap methodology using balanced repeated replication using the 500 bootstrap weights provided by Statistics Canada [36].

### Outcome—premature mortality

Individuals will be followed up longitudinally through linked population-based data (i.e., CCHS linked to CVSD) for the incidence of premature mortality. Adult premature mortality will be defined to include all deaths between the ages of 18 and 74 as registered in the CVSD. This definition aligns with the Canadian Institute of Health Information [3], which is consistent with the definition adopted in reporting of premature mortality in other industrialized nations [4–6, 37]. Respondents will be followed for a maximum of 5 years from the date of the CCHS interview (i.e., the index date) until the earliest of premature mortality, age 75 years, or end of study follow-up (December 31, 2017).

### Sample size

We anticipate the derivation cohort to consist of 329,000 respondents and the validation cohort to consist of approximately 310,000 respondents, respectively. As per CCHS sampling methodology [33], we expect there to be an approximately equal number of males and females among the derivation and validation cohorts. We anticipate approximately 12,000 premature deaths in both the derivation and validation cohorts combined with a slightly higher number of premature deaths attributable to males than females [34]. In an effort to minimize overfitting and to ensure precise estimation of key parameters in PreMPoRT, we calculated the minimum sample size necessary following the criteria proposed by Riley et al. To calculate sample size, we specified the prevalence of the outcome in our population, the number of candidate predictor variables, shrinkage (default, 0.90) and the expected model performance in terms of overall model fit (*R*^2^) [38, 39]. Using the *c*-statistic for sex-specific models in a prior population-based Mortality Risk Prediction Tool (MPoRT), we derived PreMPoRTs anticipated Cox–Snell *R*^2^. We used the R package *pmsampsize* to compute the minimum sample size to be 6933 and 8009 for the male and female models, respectively. Our expected sample sizes are well above these minimum values.

### Statistical analysis plan

The proposed analytic plan was supported by the guidelines provided by Harrell [40] and Steyerberg [41]. We have specified the analytic plan in advance of model fitting and exploration of relationships between predictor variables and the outcome. Statistical overfitting represents a concern when developing prediction models, which occur when a model captures nuances of the development data that do not appear in other applications [40, 41]. In this situation, the reliability or calibration of the model is affected and it is likely to perform poorly in other populations. Given the goal is to generalize our predictive model to Canada to help inform population-wide intervention efforts, it is important to prevent overfitting. Therefore, this study protocol is presented to improve the transparency of research, to reduce bias, and to enhance replicability of the study [42]. This study protocol has been guided by the recommended checklist of items (TRIPOD) for multivariable predictive models and will form the basis for reporting of our model estimation results [42, 43].

This prespecified analytic plan was developed with the understanding that PreMPoRT will be used by knowledge users (e.g., regional health authorities, public health departments, policymakers, and other health system decision-makers), and therefore, we made efforts to formally incorporate considerations related to the practical application and user experience of PreMPoRT. Specifically, in order to enhance usability, we plan to ensure that inputs of the model are readily available using population data that is accessible by our intended users, that the interpretation of results is meaningful across the Canadian population and by important sub-groups (i.e., socioeconomic groups), and that the model can be consistently applied across time and geography. To that effect, practical considerations and consultation with knowledge users will inform model development including areas such as predictor selection, operationalization of the model, approach to handling missing data, model specification, model estimation, model validation, and model presentation. All analyses will be conducted using SAS V.9.4. and Harrell’s *HMisc* [44] and *rms* package of functions in R, among others [45].

### Identification of predictive variables

Predictor variables were identified through screening available data collected across CCHS cycles and provinces in combination with a review of existing literature for the association with premature mortality. Additional candidate predictor variables were selected through consultation with knowledge users and our group’s previous experience developing predictive algorithms [28, 46–51]. At this stage, predictors were excluded as a result of narrow distribution or limited variability. Additionally, variables were excluded when redundancy in the information contained in the predictor was observed. A total of thirty-seven predictor variables were identified, including four health behaviors, eleven sociodemographic characteristics, seventeen chronic conditions, and five area-based measures.

Four of the area-based measures are from the Canadian Marginalization Index (CAN-Marg) which was developed using iterative factor analysis of Census data collected at the area-level [52]. CAN-Marg is an index with four domains of sociodemographic characteristics including residential instability, material deprivation, dependency, and ethnic concentration [52]. The fifth area-based measure is a binary indicator of rurality using population counts from the nearest census [53]. Information collected in the CCHS that pertains to health behaviors will be used to create summary predictors for each risk factor (further outlined below in the “Coding and cleaning of predictor variables” section). Consistent with our prior work in prediction modeling, a validated BMI correction equation will be applied to reduce bias in self-reported height and weight [54]. We will consider interactions with age and will be mindful of the possibility for interaction terms to increase over-fitting [46].

### Coding and cleaning of predictor variables

Prior to examination of predictor-outcome relation, data cleaning and predictor variable coding will be completed. Histograms and box plots will be created to graphically represent the data and to visualize the width of distributions and examine values outside of a reasonable range. We will focus on minimizing loss of predictive information, where we will pre-specify BMI as continuous using restricted cubic splines and knots placed based on the World Health Organization classifications [55]. Derived predictors will also take into consideration how our group has defined predictors in prior population-based prediction models [28, 46–51]. We may also group or exclude candidate predictors based on the categories with a small proportion of respondents (i.e., < 5%) to avoid instability in regression modeling. Consistent with previous model development approaches, we will derive some related predictors based on a combination of survey questions in the CCHS. For example, smoking status will be defined based on questions that probe whether a respondent has smoked at least 100 or more cigarettes in their lifetime, whether the respondent currently smokes cigarettes, how many cigarettes are smoked each day/month, and whether the respondent has previously quit smoking. Additional details about the questions and response options used to collect information about health behaviors including alcohol consumption, fruit and vegetable consumption, cigarette smoking, and leisure-time physical activity can be found in Supplementary Table 1, Additional file 1.

### Approach to missing data

To avoid limitations associated with available case analysis [41], we will use multiple imputation methods to assign missing values on select predictor variables, using the approach recommended by Rubin and Schenker [56]. In our experience using the CCHS for predictive modeling [28, 46–51], no predictor variable is expected to have ≥ 10% missingness in the six combined cycles. The statistical approach used for imputation will include the full set of predictor variables, time to event and censoring variables, and secondary variables (i.e., variables that are not candidate predictors but may be valuable in producing imputed estimates). Five copies of the multiple imputed data sets will be used and combined using Rubin’s rules to provide an overall estimate for each regression coefficient or measure of interest (e.g., c statistic, calibration plot) that takes into account the uncertainty in the imputed estimates [57]. We will implement multiple imputation using the multivariate imputation by chained equations (mice) algorithm in R [56, 58] and include the Nelson–Aalen estimator of the baseline hazard for premature death [59].

### Model estimation

The probability of 5-year premature mortality will be assessed from the interview date until the incidence of premature mortality, censoring for death, or end of the follow-up period. The initial models will be estimated using the Weibull accelerated failure time model, a type of parametric regression that can also be specified as a proportional hazards model. We chose this model for several reasons: (1) the user can predict survival time for a variety of follow-up periods; (2) the full maximum likelihood can be used for parameter estimation; and (3) parameter estimates provide intuitive estimates of effect [60]. In addition, our group’s previous experience with the development and validation of population-based prediction models [28, 46–51] demonstrates that the Weibull model performs well for prediction tasks.

To assess the adherence to parametric assumptions of the Weibull model, we will use stratified Kaplan–Meier curves whereby a graph of the log survival time versus log [–log(KM)] will display parallel and linear lines if the model is appropriate [60]. The proportional hazards assumption will also be checked for each predictor using stratified plots of the log cumulative hazard and examination of Schoenfeld residuals. In the case where the Weibull distribution results in inaccurate predictions and/or model convergence concerns given that the hazard function of the model contains a different shape (e.g., U-shape, J-shape), alternative model specifications and flexible parametric survival model will be examined. Model overfitting will be assessed based on the log-likelihood ratio *X*^2^ statistic for the full model, with evidence of overfitting being a shrinkage < 0.90. Before finalizing our model, we will also assess the fit of the Royston–Parmar model, which allows baseline hazards to be modeled more flexibly. We do not anticipate this model to offer advantages in this context based on our previous experience with other population risk outcomes for chronic disease, where we observed no advantage given the linear baseline hazard [61, 62]. It also offers a significant drawback for users of the model, who will not have the ability to re-estimate the baseline hazard given that they do not have access to the linked data [61, 62]. Population-level survey weights provided by Statistics Canada will be used to allow estimates to be representative of the population. The model will then be used to derive a survival risk function to predict the probability of premature mortality during a 5-year follow-up period.

### Model specification

Separate models will be derived using the pre-specified forms of predictor variables identified in Table 1 for men and women. As recommended by Harrell, continuous predictor variables will be modeled in a flexible manor using restricted cubic splines with the knots placed at fixed quantiles of the distribution, facilitating flexibility and increased stability in the tails of the function [40]. During the model building process, alternative forms of pre-specified candidate variables may be explored. For example, we intend on exploring physical activity as a continuous predictor (i.e., average daily metabolic equivalent of task as specified in Table 1) and as an ordinal predictor (4 quartiles of physical activity). The continuous and categorical form of the predictor will be compared in terms of measures of predictive performance including overall fit, discrimination, and calibration in addition to the information criterion (e.g., AIC and BIC). The variable form that improves the overall model fit will be selected, and the continuous and centered form of both categorical and continuous predictors will be used. Two-way predictor interactions between age and other variables will be explored. The initial model will be fit using the pre-specified forms of the predictors which have 77 degrees of freedom (Table 1).

**Table 1:** Pre-specification of PreMPoRT predictive variables

Variable category	Variable	Definition	df^1^
Health behaviors	Alcohol consumption		3
	Non-drinker	No alcohol consumption in the last 12 months or drink frequency fewer than once a week	
	Light drinker	Alcohol consumption frequency at least once a week and 0–2 (females) or 0–3 (males) drinks in the previous week	
	Moderate drinker	3–14 (females) or 4–21 (males) drinks in the previous week	
	Heavy drinker	≥ 14 (females) or ≥ 21 (males) drinks in the previous week, or binging behavior on a weekly basis (≥ 5 drinks on any occasion)	
	Daily fruit and vegetable consumption		2
	Low consumption	0 to less than 3 times daily	
	Medium consumption	3 to less than 6 times daily	
	High consumption	6 or more times daily	
	Cigarette smoking		4
	Non-smoker	Never a smoker or former occasional smoker with < 100 lifetime cigarettes	
	Former heavy smoker	Former smoker [≥ 1 pack (25 cigarettes)/day]	
	Former light smoker	Former smoker [< 1 pack (25 cigarettes)/day]	
	Heavy smoker	Current smoker [≥ 1 pack (25 cigarettes)/day]	
	Light smoker	Current smoker [< 1 pack (25 cigarettes)/day]	
	Leisure physical activity (kcal/kg/day)	4 knot restricted cubic spline	3
Sociodemographic characteristics and self-perceived measures	Age (years)	5 knot restricted cubic spline	4
	Ethnicity		1
	White		
	Non-white		
	Immigration status		2
	Canadian born		
	Recent immigrant	Immigrated < 10 years	
	Non-recent immigrant	Immigrated ≥ 10 years	
	Household income		4
	Quintile 1	Lowest 20%	
	Quintile 2		
	Quintile 3		
	Quintile 4		
	Quintile 5	Highest 20%	
	Home ownership		1
	Yes		
	No		
	Education		2
	Less than secondary school graduation		
	Secondary school graduation		
	Post-secondary education (complete and partial)		
	Marital status		2
	Single never married		
	Domestic partner (married/common law)		
	Widowed/separated/divorced		
	Body mass index (BMI) (kg/m^2^)	5 knot restricted cubic spline	4
	Self-perceived general health		4
	Excellent		
	Very good		
	Good		
	Fair		
	Poor		
	Self-perceived life stress		4
	Not at all		
	Not very		
	A bit stressful		
	Quite a bit		
	Extremely stressful		
	**Self-perceived community belonging**		3
	Very strong		
	Somewhat strong		
	Somewhat weak		
	Very weak		
**Chronic conditions**	**Physician diagnosed chronic conditions**		17
	Including asthma, arthritis, back problems, high blood pressure, migraines, emphysema, chronic obstructive pulmonary disease, diabetes, heart disease, cancer, intestinal ulcers, stroke, urinary incontinence, bowel disease, mood disorder, or anxiety disorder	Yes; no for each individual chronic condition	
Area-based measures	Rurality		1
	Population center	Population of at least 1000 and a density of ≥ 400 people per square kilometer based on current census population counts	
	Rural area	Population concentration or densities below the urban threshold based on current census population counts	
	Material deprivation		4
	Quintile 1	Least deprived	
	Quintile 2		
	Quintile 3		
	Quintile 4		
	Quintile 5	Most deprived	
	Ethnic concentration		4
	Quintile 1	Least concentrated	
	Quintile 2		
	Quintile 3		
	Quintile 4		
	Quintile 5	Most concentrated	
	Residential instability		4
	Quintile 1	Least unstable	
	Quintile 2		
	Quintile 3		
	Quintile 4		
	Quintile 5	Most unstable	
	Dependency		4
	Quintile 1	Least dependent	
	Quintile 2		
	Quintile 3		
	Quintile 4		
	Quintile 5	Most dependent	

^1^Degrees of freedom

The model building approach will include all a priori predictors (Table 1) with a step-down model selection that includes confirmation (i.e., assessment of impact on predictive performance) at each step. The overall fit of the full model will be assessed according to model fit statistics and overall measures of predictive accuracy. Variables will be removed from the model, one set at a time. To verify if variable exclusions were appropriate, variables omitted in previous model building steps will be re-added to the model to verify whether the initial exclusion was justified. In addition to the use of more traditional methods of model building, we will also verify our model building approach using the least absolute shrinkage and selection operator (LASSO) which may assist in avoiding model overfitting [41].

### Model validation

For internal validation, we will apply a bootstrap validation in the development cohort as an internal validation approach to generate measures of model performance [40, 41, 63], which we have used for internal validation in other population risk models [64]. Bootstrap samples using 500 bootstrap repetitions [41] will be drawn, and bootstrap models will be developed on each sample. Each bootstrap model will then be applied to the original data, and the difference in model performance (i.e., discrimination and calibration) between the bootstrap models and the original development model can be averaged to adjust for the expected optimism of the model. For example, using bootstrap validation, we will present optimism-corrected performance metrics (i.e., optimism-corrected *R*^2^ and optimism-corrected *c*-statistic) as recommended by Steyerberg [41]. Additionally, the degree of model overfitting will be quantified using the heuristic shrinkage estimator, which is based on the log-likelihood ratio *X*^2^ statistic of the fitted model. The model will be adjusted for overfitting if the shrinkage is below 0.9; however, if the estimated shrinkage is greater than 0.9 and the model performs poorly, then alternative data reduction approaches will be considered [40]. Following internal model validation, the model will be externally validated in the combined CCHS cycles 2007–2008, 2009–2010, and 2011–2012 and will be evaluated according to measures of overall predictive accuracy, discrimination, and calibration. The full Canadian dataset will be used to derive final regression coefficients, in an effort to optimize the sample size and follow-up period with the same predictor variables and form as specified in the derivation model. This approach is recommended as differences in regression coefficients between the development and validation dataset are expected to be small and using the full dataset facilitates stability in regression estimates [41].

### Assessment of model performance

The overall predictive performance in both the derivation and validation cohorts will be evaluated and reported using overall measures of predictive accuracy, discrimination (how well a model can differentiate between low- and high-risk respondents), and calibration (agreement between observed and predicted outcomes). Specifically, measures of overall accuracy will be assessed using Nagelkerke’s *R*^2^ and Brier score. Discrimination will be assessed with Harrell’s concordance statistic, with confidence intervals calculated using bootstrapping procedures with 10 iterations. In predicting binary outcomes such as premature mortality, the concordance statistic is equal to the area under the receiver operating characteristic (ROC) curve. The calibration of our model is of primary importance; therefore, calibration will be optimally assessed through graphical inspection of calibration plots with observed plotted against predicted risk.

Steyerberg [41] and Cook [65, 66] suggest that calibration is of primary importance in prediction modeling and recalibration tests (e.g., calibration-in-the-large and calibration slope) should be routinely assessed during model performance evaluation. Therefore, calibration plots will be studied at fixed points in time with observed survival compared to the mean predicted survival among groups of respondents using the Kaplan–Meier method. Overall calibration can be evaluated through Wald or likelihood ratio test to determine if there is derivation from perfect calibration (i.e., slope of one) with the calibration plot displaying the combined effect of systematic differences between the new data and the model development data and overfitting from the effects of predictor variables. Further, calibration in the small will be assessed for predefined subgroups (i.e., provinces and rural/urban geography) of importance to knowledge users and decision-makers, for example by defined age or sociodemographic groups. Consistent with guidance [67] and previous studies [64], we define adequate calibration as a relative difference of < 20% between observed and predicted risk for sub-groups with at least a 5% prevalence of premature mortality.

### Model presentation

The final regression model for PreMPoRT consisting of both the derivation and validation sample will be presented using beta estimates, hazard ratios, and 95% confidence intervals. Model presentation will consist of the regression formula which will form the foundation for all Internet-based implementation and integration. Visualizations of the tool will be generated to help with knowledge translation approaches and to improve model literacy among non-technical users.

## Discussion

We have developed this protocol in consultation with our existing partnerships in local Public Health Departments and will continue to ensure that PreMPoRT meets the needs of the knowledge user as we engage stakeholders at several stages of development. This integration process will enable PreMPoRT for applications in diverse settings and regions across Canadian provinces with the support of our knowledge users to assist in predicting the incidence of premature mortality. PreMPoRT will be used to produce estimates of future premature mortality, to assess the contribution of specific risk factors to overall population risk, and will assist in identifying groups at an elevated risk of premature mortality. We anticipate that this information will be particularly useful for planners and decision-makers when considering intervention approaches to reduce inequities in premature mortality.

### Limitations

One notable limitation of PreMPoRT is that while the tool will be representative of most of the Canadian population (98%), some groups are not covered by the CCHS sampling methodology including Indigenous people living on First Nation reserves. This is important given that these population have different risk of premature mortality than the general population [68]. An additional concern related to the development of predictive algorithms, such as PreMPoRT, include the potential for overfitting and type 1 error, which may occur if the association between the predictor and outcome influence whether the predictor is included and how the model is developed. In an effort to reduce this risk, we have prespecified our analytic plan, as presented in this protocol. Due to the use of self-reported nature of predictors captured at a single point in time, there is potential for misclassification error, both systematic and non-directional. Despite this limitation, we have found self-reported data to be robust and accurate for prediction of other outcomes, including diabetes [69], obesity [51], all-cause mortality [28], multiple chronic diseases [64], and high-cost users [70]. Finally, we anticipate that further model updating may be needed to account for the potential change in the baseline survival in other countries, which we will include in our recommendations.

## Conclusions

To the best of our knowledge, PreMPoRT will be the first population-based regression model to predict the incidence of premature mortality. We anticipate that the tool will assist in meeting the needs of knowledge users who value evidence-informed decision-making to assist with population-level planning. This research demonstrates a mechanism whereby routinely collected population-level data can be used to inform more equitable and impactful population health strategies.

## Supplementary information

**Additional file 1.** Title: Lifestyle risk factor questions from the Canadian Community Health Survey. Description: Canadian Community Health Survey questions and response options that are used to create the summary health behavior variables.

## Data Availability

The data used to generate the study cohort are available only through one of the Statistics Canada Research Data Centres, but restrictions apply to the availability of these data, which were used under license for the current study, and so are not publicly available. Data are however available from the authors upon reasonable request and with permission of the Research Data Centre.

## Abbreviations

PreMPoRT: Premature Mortality Population Risk Tool
CCHS: Canadian Community Health Survey
CVSD: Canadian Vital Statistics Database
BMI: Body mass index
TRIPOD: Transparent reporting of a multivariable prediction model for individual prognosis or diagnosis
Can-MARG: Canadian Marginalization Index
df: Degrees of freedom
AIC: Akaike information criterion
BIC: Bayesian information criterion
LASSO: Least absolute shrinkage and selection operator
